# The Ripple Effect: How Hallux Valgus Deformity Influences Ankle and Knee Joint Kinematics During Gait

**DOI:** 10.3390/bioengineering12070744

**Published:** 2025-07-08

**Authors:** Longzhou Hua, Chenglin Wu, Ye Luo, Longxiang Li, Mingwei Liu, Aoqing Huang, Fangfang Li, Zhongmin Shi, Shaobai Wang

**Affiliations:** 1 School of Exercise and Health, Shanghai University of Sport, 200 Hengren Road, Shanghai 200438, China; 2 Department of Orthopedics, The Sixth People’s Hospital, Shanghai Jiaotong University School of Medicine, Shanghai 200233, China

**Keywords:** hallux valgus, 3D, motion analysis, gait, 6-DOF

## Abstract

Hallux valgus (HV) is described as a lateral deviation of the great toe at the first metatarsophalangeal joint (MTP), which is a very common foot deformity in the clinic. This deformity extends beyond localized foot mechanics to affect the entire lower extremity kinetic chain, potentially increasing dynamic instability during locomotion. This study aimed to characterize the kinematics of ankle and knee joints during walking in HV patients compared to controls. In total, 23 patients with bilateral HV and matched healthy controls were recruited. The 6-DOF kinematics data of ankles and knees were collected using a joint motion function analysis system while level walking at adaptive speed. HV patients demonstrated significant kinematic alterations in the ankle joint at IC, including decreased varus by 2.87° (*p* < 0.001), decreased internal rotation by 1.77° (*p* = 0.035), and decreased plantarflexion by 4.39° (*p* < 0.001) compared with healthy subjects. Concurrent compensatory changes in the knee joint included increased varus rotation by 1.41° (*p* = 0.023), reduced anterior translation by 0.84 mm (*p* < 0.001), and increased lateral translation by 0.26 mm (*p* = 0.036). HV patients showed increased ankle dorsiflexion of 3.61° (*p* = 0.06) and decreased ankle internal rotation of 2.69° (*p* = 0.043), with concurrent increased knee internal rotation of 2.59° (*p* = 0.009) at SPF. The ripple effect during walking in the HV population may elevate the risk of knee pathologies. These findings may inform both conservative management strategies and post-surgical rehabilitation regimens.

## 1. Introduction

Hallux valgus (HV) is one of the most prevalent forefoot deformities in clinical practice, affecting approximately 19% of the global population, with a pronounced female predominance (up to 90% of cases) [1]. Beyond its local effects on the first metatarsophalangeal joint (MTP), HV has emerged as a potential contributor to broader biomechanical dysfunction throughout the lower extremity kinetic chain, with implications for fall risk, dynamic instability, and the development of secondary pathologies in proximal joints [1,2]. The disruption of the transverse arch and abnormal pressure distribution across the forefoot fundamentally alter foot mechanics during weight-bearing activities [3,4]. A ripple effect or compensatory mechanism can be demonstrated if the related deviations are further increased in response to more constraining conditions [5]. These structural changes in patients with HV compromise the foot’s natural cushioning function, potentially transferring mechanical stresses proximally to adjacent joints such as the knee. Indeed, case studies suggested that HV contributes to knee cartilage damage, ligament injury, or knee osteoarthritis (OA), indicating that HV pathology not only affects foot anatomy but also creates a ripple effect in proximal joints [6].

Previous studies on patients with HV have primarily focused on local pathological changes in the MTP joint and the effects of surgical interventions (such as first metatarsal osteotomy, McBride procedure, and Akin osteotomy) on ankle joint function [2,7,8]. Biomechanically, research in patients with HV has focused on the measurement of plantar pressure distribution [9,10,11]. A more comprehensive understanding of the compensatory changes in response to HV deformity would be tough to acquire without evidence of the kinematic changes in the adjacent segment. Only a few small-sample studies have reported the effect of HV deformity on proximal kinematics [4,12]. Shih et al. [4] found that knee abduction decreased during the terminal stance phase in a study involving 12 women with bilateral HV, a biomechanical change that may increase pressure on the medial knee compartment. Kozáková et al. [12] identified increased peak knee extension at the end of the swing phase in another study with a limited sample size (*n* = 6 HV patients), but only changes in the sagittal and frontal planes of the knee joint were addressed. The smaller sample sizes or different symptoms in HV patients may contribute to differences in the statistical power and generalizability of findings. Additionally, more comprehensive spatial (three-dimensional) 3D motion analysis based on dual-head infrared stereo cameras has shown some promise due to its time-saving, convenience, and accuracy in clinical analysis over the last decade or so of studies [13,14].

This study aimed to examine the ripple effect of kinematic alterations of bilateral HV patients in the ankle and knee joint across six degrees of freedom (6-DOF) in 3D space while level walking at a self-selected speed at clinical application using a joint motion analysis system. We hypothesized that patients with HV would demonstrate coupled movements of both joints during gait, decreased ankle varus, and internal rotation concomitant with increased knee varus rotation.

## 2. Materials and Methods

### 2.1. Participants

Sample size determination was conducted using a pilot study of five subjects (10 ankles and knees) per group with G*Power software (version 3.1.9.7) using the ‘Two Independent Means’ module. Flexion angle at IC served as the primary kinematic parameter. Pilot data revealed mean flexion values of −3.09° (HV group) and −7.20° (Control group). With the power set at 0.9 and the significance level set at α = 0.05, the analysis indicated that 26 knees per group would provide adequate power to detect between-group kinematic differences. A total of 23 patients with bilateral HV from the Foot and Ankle Surgery Centre and cases of HV accompanied by flatfoot were not included due to ongoing controversy. Therefore, the current study sample (46 ankles and knees per group for both the HV and Control groups) exceeded this requirement. In total, 23 patients (46 ankles and knees collectively) diagnosed with bilateral HV (20 females and 3 males; mean age 36.9 years; mean height 161.8 cm; mean body mass index 21.85 kg/m^2^) were studied alongside 23 healthy volunteers matched for their condition. All patients were diagnosed with HV deformity via weight-bearing CT, with severe HV deformity characterized by a valgus angle exceeding 40 degrees and an intermetatarsal 1–2 angle surpassing 16 degrees [2]. Injuries or conditions of the lower limb joints, excluding HV, were also excluded through ankle internal and external rotation tests, knee drawer tests, and crush and grind tests. The inclusion criteria for volunteers included the absence of significant trauma to the lower extremities, a history of surgical procedures, and neuromusculoskeletal disorders. This study was approved by the Institutional Review Board of our university.

### 2.2. Instrumentation

**Figure 1:**
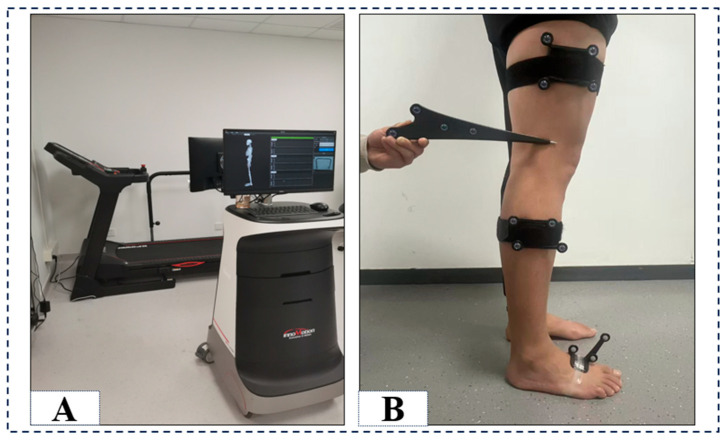
The instrumentation configuration (**A**) and computed spatial 3D positions of the knee and ankle joints, illustrated with the probe directed towards the lateral epicondyle of the femur (**B**).

The study employed a joint motion analysis system (Opti-Knee, Innomotion Inc., Shanghai, China) to gather data on ankle and knee joint kinematics (Figure 1A). The system was dynamic, real-time, three-dimensional, and objective, with a footprint of only 4.0 m × 2.0 m × 2.5 m. Participants conducted this experiment on a bidirectional treadmill (Innomotion Inc., Shanghai, China). Two pairs (four) of infrared optical trackers were affixed to the thighs and calves on each side of the subject, positioned 8 cm above the lateral femoral epicondyle, 3 cm below the fibular head of the calf, and on the foot, while the 3D trajectories of the four corresponding reflecting spheres (OK-Marquer; Innomotion Inc.) were recorded by a dual-headed infrared stereoscopic camera operating at a frequency of 60 Hz and achieving a tracking accuracy of 0.3 mm RMS [15]. The handheld probe comprises four markers that reflect infrared light to identify osseous landmarks and determine the geometric position of the two joint complexes [16,17]. It was performed to analyze the gait cycle by an integrated synchronous high-speed camera and compute the 3D kinematic parameters of biarticulated joints using customized software (Optimum, Innomotion Inc.). The repeatability of this joint motion function analysis system is less than 0.9 mm for translation and less than 1.3° for rotation [16].

### 2.3. Experimental Protocol

An experienced investigator utilized the handheld probe to delineate bony landmarks, including the greater trochanter of the femur, the medial and lateral epicondyles of the femur, the medial and lateral malleoli, and the second metatarsal, as well as the neutral ground position, for static alignment and to establish baseline anatomical relationships between the femur, tibia, and ankle complex, thereby determining the axis of motion of the knee joint [16,17]. Following a warm-up of 5 min to replicate their daily normal over-the-ground gait on a treadmill [18], binocular stereo infrared lenses captured kinematic data of their knee joints in 3D and 6-DOF at a rate of 60 frames per second for 15 s. Subjects walked at an adaptive speed on a bidirectional treadmill, ensuring consistency in their bidirectional walking speed to minimize errors.

### 2.4. Date Collection

**Figure 2:**
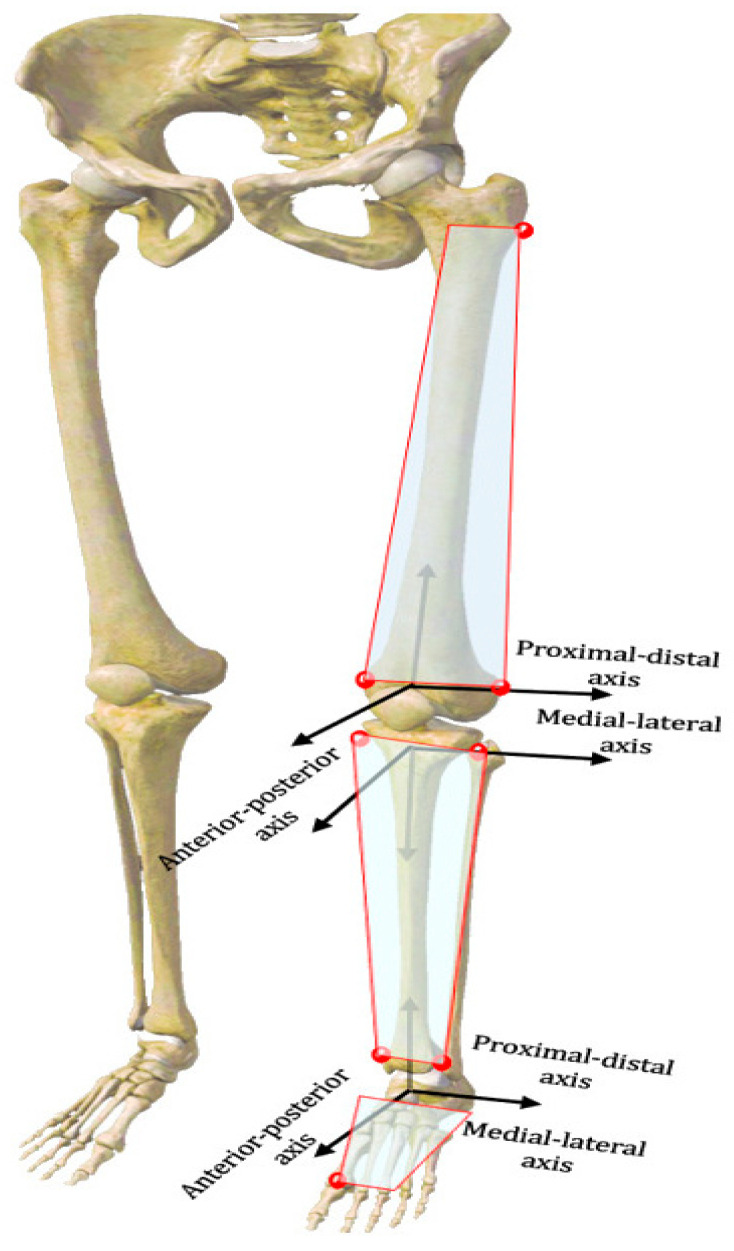
Definition of the localized coordinate system for the femoral and tibial and ankle complexes.

The 3D spatial coordinates of the knee and ankle joints were computed for each walking frame, using the geometric relationships among the anatomical positions of the femur, tibia, and ankle complexes, as established by the handheld probe and the rigid tracking plates of the femur, calf, and ankle [16,17] (Figure 1B). Various 3D joint kinematic parameters were measured, including plantarflexion-dorsiflexion, varus-valgus and internal-external rotation, and translation in three different planes: sagittal, horizontal, and frontal (Figure 2). Rotational analysis determined angular changes by comparing the proximal reference system orientation against the distal reference system across three anatomical planes: medial–lateral, anterior–posterior, and proximal–distal directions using Euler angle decomposition. To eliminate dependency on rotation order, a projection-based approach was employed for characterizing joint angular motion. Translational motion was assessed by computing the relative displacement between the coordinate system origins of the proximal bone, with the results expressed within the distal bone’s reference frame [14]. The subjects’ gait data were standardized to 100 points (0–100% heel strike to next heel strike) per gait cycle, which were subsequently partitioned into the stance phase and swing phase [14]. We exclusively analyzed the stance phase of the gait cycle, as it constitutes the primary segment of the cycle. The knee 6DOF kinematics during the initial contact (IC), stance peak flexion (SPF), and stance peak extension (SPE) phases, as well as the ankle joint’s one-to-one kinematics, were examined during gait.

### 2.5. Statistical Analysis

Statistical analyses were performed by SPSS version 27.0 (IBM, Armonk, NY, USA). Before statistical analyses, all data were of a normal distribution and Chi-square-tested. Independent *t*-tests expressed normal-distributed continuous variables as means (with standard deviations). Otherwise, the variables were median (interquartile range, IQR) and tested with the Wilcoxon signed rank test. *p* < 0.05 was considered significant.

## 3. Result

### 3.1. Characteristics of the Participants

In total, 23 bilateral HV patients and 23 volunteers participated in this study. Table 1 shows the mean age, gender, height, weight, and BMI of both groups, obtained from questionnaires or direct measurements.

No statistically significant differences were observed between the two subject groups regarding age (*p* = 0.783), gender (*p* = 0.968), height (*p* = 0.344), weight (*p* = 0.212), and body mass index (*p* = 0.378).

### 3.2. 3D Kinematic Curves of the Ankle and Knee Joints

**Figure 3:**
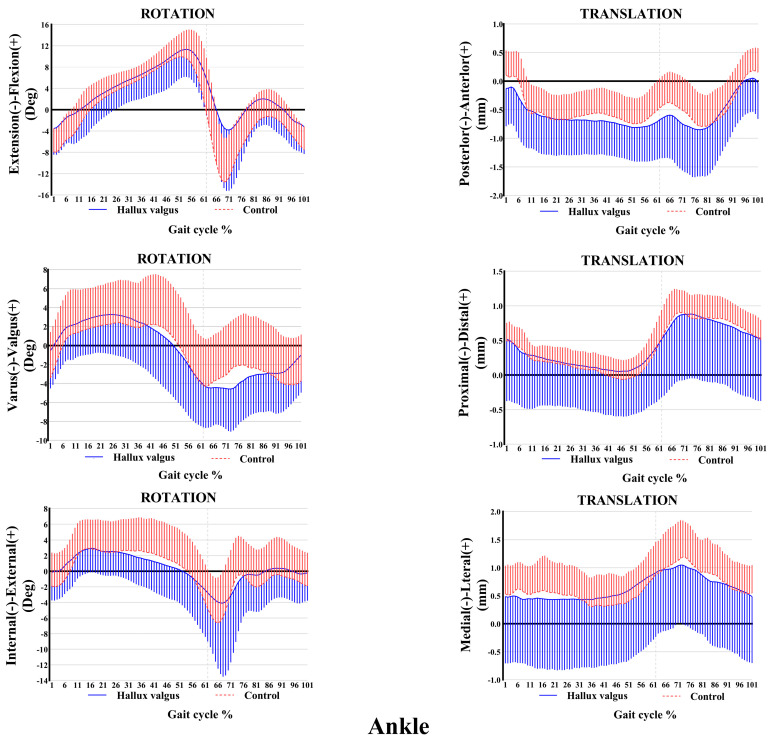
Ankle kinematic features of 6-DOF at adaptive speed on a treadmill. The ensembles of each participant were normalized from the heel strike to the next heel strike of the same foot as a gait cycle. The solid curves and the lines below them represent the mean and the standard deviation of the gait cycle for the HV group, while the dashed curves and the lines above them are for the control group. Deg: degree; mm: millimeter.

**Figure 4:**
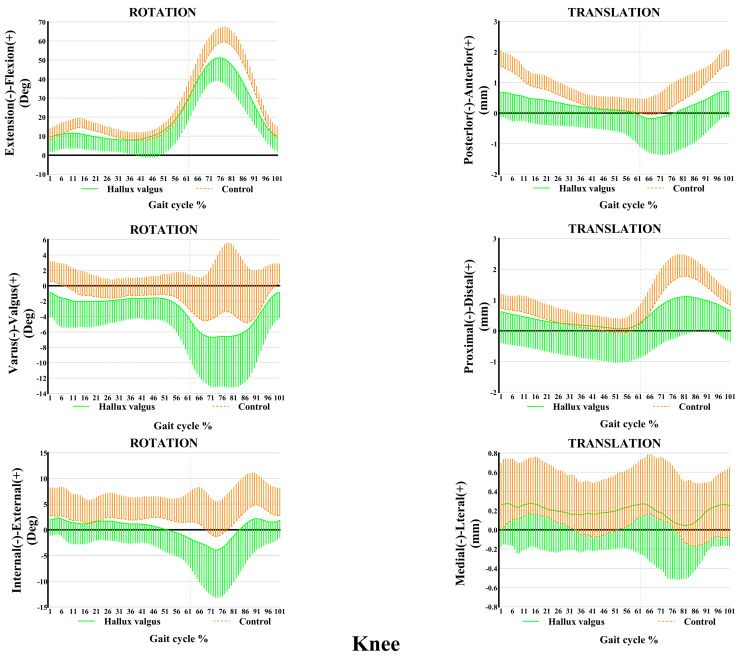
Knee kinematic features of 6-DOF at an adaptive speed on a treadmill. The ensembles of each participant were normalized from the heel strike to the next heel strike of the same foot as a gait cycle. The solid curves and the lines below them represent the mean and the standard deviation of the gait cycle for the HV group, while the dashed curves and the lines above them are for the control group. Deg: degree; mm: millimeter.

The ankle and knee kinematic features in patients with HV during the adaptive velocity gait cycle are shown in Figure 3 and Figure 4, respectively. HV populations exhibit contrasting kinematic profiles in adjacent joints as a compensatory mechanism. Comparing the healthy subjects, less plantarflexion (~0.45°–4.63°) and more valgus rotation (~0°–2.86°) and posterior translation (~0 mm–0.20 mm) of the ankle joint were seen during the stance phase in patients with HV, whereas only slight changes appeared in other directions of motion. Valgus rotation increased mainly during the first two-thirds of the stance phase, whereas the change in plantarflexion and posterior translation occurred during the entire stance phase. Additionally, increased knee inversion (~0.40°–1.67°) and internal rotation (~0°–3.30°) were seen in patients with HV during the stance phase of the gait cycle. Regarding translations, less anterior (~0 mm–0.84 mm) and more lateral translations (~0.1 mm–0.28 mm) were seen during the entire stance phase of the gait, whereas no significant proximal-distal difference appeared during the stance phase.

### 3.3. Comparison of 6-DOF of Double Joint Kinematic Parameters at Key Event

The biarticular kinematic parameters at the critical event of gait in HV patients and healthy volunteers are shown in Table 2. Patients with HV exhibited decreased varus rotation by 2.87° (SD 0.92, (95% CI −4.68 to −1.03), *p* = 0.003), internal rotation by 1.77° (SD 0.83 (95% CI −3.41 to −0.13), *p* = 0.035), and plantarflexion by 4.39° (SD 0.98, (95% CI −6.33 to −2.45), *p* < 0.001) of the ankle joint at IC. Additionally, they exhibited increased varus rotation by 1.41° (SD 0.61, (95% CI 0.20 to 2.62), *p* = 0.023), lateral translation by 0.26 mm (SD 0.12, (95% CI 0.02 to 0.50), *p* = 0.036), and anterior translation by 0.84 mm (SD 0.14, (95% CI −1.13 to −0.56), *p* < 0.001) of the knee joint compared with healthy subjects at IC.

Patients with HV exhibited a notable increase in ankle dorsiflexion (from 2.29° (IQR 7.12) to 5.90° (IQR 12.18), *p* = 0.006) and a decrease in internal rotation (from−5.05° (IQR 7.82) to −2.36° (IQR 6.52), *p* = 0.043) during the gait cycle, and their knees demonstrated an increase in internal rotation (from 1.37° (IQR 7.92) to −1.22° (IQR 6.04), *p* = 0.009) at SPF, which are elaborated on in Table 3. The differences were not statistically significant between the two populations at SPE.

## 4. Discussion

This study investigated alterations in ankle and knee kinematics during level walking at a self-selected speed between patients with bilateral HV and healthy subjects. The key finding of this study was decreased ankle varus and internal rotation as well as plantar flexion in HV patients during the stance phase. Additionally, there was increased knee varus rotation, decreased anterior translation, and increased lateral translation. These results suggest a ripple effect in HV patients, which has an adverse impact on adjacent joint function.

Deformity of the first MTP alters the overall function of the foot, which is vital to the biomechanics of the foot [19]. Our analysis indicated that patients with HV exhibited more ankle dorsiflexion and valgus alignment at IC as well as SPF during the stance phase compared to the healthy subjects, which were comparable to previous results [20,21]. Kawakami et al. documented a 24% decrease in plantarflexion and an 18% increase in hindfoot valgus, respectively, during the stance phase by comparing foot joint coordination and variability between 25 patients with HV and healthy women according to a multi-segment foot model across two investigations [20,21]. Karl et al. did not find differences in hindfoot kinematics between 19 patients with HV and healthy ambulators, which may be due to the use of different foot models between researchers [8]. The reduction in ankle plantarflexion at IC compromises the foot’s natural shock absorption mechanism, reducing the available range of motion for controlled plantarflexion during the stance phase, potentially transmitting increased mechanical stress to proximal joints [4,22]. In addition, muscle imbalance in abductor and adductor muscles was cited as a major factor in the production of HV [23]. Muscle imbalance affects the function of the entire intrinsic foot muscles, with one study surfacing reduced tibialis anterior muscle activation in elderly people with HV during walking [24], and in another investigating the movement of young HV sufferers from sitting to standing, no differences in muscle synergy with the healthy controls were found, which contradicts the current study of reduced ankle plantarflexion [25], which may be due to the different performance due to the decline in muscle strength associated with aging.

It is assumed that excessive valgus rotation of the ankle lowers foot rigidity by obstructing the midtarsal locking mechanism and causes instability in the forefoot during the kick-off phase [22]. As the abnormal linear alignment of the MTP and the arch structure is affected, this leads to the distribution of the repeated forefoot pressure during the terminal stance [3,26]. Conservative treatments (incorporating foot orthotics and minimalist shoes) could achieve the forefoot pressure redistribution, decreasing loading beneath the hallux and MTP regions and improving dynamic stabilization, which prevents further exacerbation of surrounding structures [27]. For example, new smart orthopedic insoles with embedded wearable pressure sensors in orthopedic insoles combined with integrated electronic systems have demonstrated great potential for monitoring plantar pressure redistribution in HV patients at different wearing time periods due to their small size and portability [28]. Additionally, finite element analysis indicated the first metatarsal osteotomy may also help maintain normal plantar force pressure distributions and lower limb stability [29].

The pathology of the foot has been demonstrated to affect the kinematic chain during walking and lead to a change in the load on other joints, which can result in further pathology [26]. The current study showed that knee varus rotation at IC (almost entire gait) increased significantly in patients with HV compared with healthy subjects, which was comparable to previous studies. Shih et al. [4] showed increased hip internal rotation using a modeled seven-link system and knee abductor moments by inverse dynamics in patients with HV during the stance phase, which explains the increased knee varus rotation. Additionally, Kozáková et al. [12] showed a 3.97° decrease in the maximum values of knee valgus in patients with HV during the walking gait by comparing the gait of 6 female patients with asymptomatic bilateral HV and 11 healthy controls using a Vicon motion capture system. The differences in results may be due to the presence of different symptoms in patients with HV, as well as differences in age. It is well acknowledged that consistent increased knee varus moments will enhance the risk of developing OA. Wearing high-heeled shoes also showed increased knee varus moments and angles based on inverse dynamics calculations [30]. Hence, minimalist shoes instead of fashionable high-heeled shoes for patients with HV may help reduce the medial pressure of the tibiofemoral joint.

Knee kinematic changes in translation have not been reported in patients with HV. We found that, compared with healthy subjects, there was slight lateral translation (0.26 mm) in patients with HV at the IC phase. The degeneration of the medial compartment of the knee joint results from prolonged knee varus deformity and the relative laxity of the medial collateral ligament in the extended position, which diminishes its capacity to restrict knee joint medial–lateral movement [31,32]. Therefore, patients with HV may be at an elevated risk for developing medial compartment knee osteoarthritis, even in the absence of acute knee symptoms [4]. Additionally, HV patients diminish external knee rotation at the IC phase (though the difference was not statistically significant), elongating the anterior cruciate ligament (ACL) and inhibiting the anterior translation of the tibia relative to the femur. There are different anatomical morphologies for the medial and lateral femoral condyles; the lateral condyles possess greater thickness and a bigger coronal diameter, but the medial condyles exhibit an elongated anterior–posterior diameter [33,34]. This anatomy clearly illustrates that the internal rotation of the femur may result in posterior translation of the knee joint in the horizontal plane. Thus, these changes in our study may have been an adaptive response to biomechanical alterations due to deformity of the first MTP. A previous study showed that the knee typically had more internal rotation during a walking gait cycle in ACL-deficient knees compared with healthy knees [35]. For this reason, it may increase the risk of instability and injury of the knee joint in patients with HV when implement challenge motions.

Compared with healthy subjects, our analysis indicated that patients with HV increased internal rotation at the SPF phase. Extra activation of the semitendinosus in patients with HV based on muscle and kinematic synergies analysis supports this finding [24]. The kinetic chain theory posits that foot anomalies propagate to the tibia. Forefoot rotation may induce internal rotation of the tibia, necessitating compensatory internal rotation of the knee to preserve dynamic equilibrium. In support of this, Resende et al. [36] developed a wedge sandal to induce ankle valgus and observed a notable increase in knee internal rotation among their participants. Additionally, HV patients inwardly rotate the hip joint, increasing the femur angle of adduction to relieve pressure on the MPT [4]. Femoral internal rotation is a prevalent biomechanical contributor to knee injury [37]. Increased internal rotation of the knee may increase the risk of patellofemoral joint pain syndrome and non-contact injuries of the ACL [38,39]. Additionally, internal rotation of the tibia exerts mechanical compression on the medial compartment of the knee, hence exacerbating the progression of OA [40]. However, advanced measurement techniques—including 3D fluoroscopy [41] combined with non-invasive artificial intelligence (AI)-enhanced EMG signal acquisition methods [42]—are needed to quantify HV’s effects on dynamic ankle and knee joint alignment and provide more direct evidence.

The ripple effect extends proximally to the ankle and knee joints in patients with HV. These adaptations—characterized by decreased ankle varus rotation, internal rotation, and plantarflexion, together with increased knee varus rotation and lateral translations—demonstrate that HV pathology influences the entire lower extremity kinetic chain rather than remaining isolated to the forefoot. These observations carry significant clinical implications for both conservative management and surgical intervention strategies. Comprehensive rehabilitation regimens should extend beyond localized foot and MTP interventions to address the altered biomechanics at the ankle and knee [43,44,45]. For example, the integration of AI techniques, including long short-term memory (LSTM) and U-Net models, demonstrates promise for the prediction of gait kinematics and the risk of adjacent joint or ligament injury across different severities of HV [46]. Recent studies have demonstrated that LSTM networks enable the forecasting of kinematic deviations by capturing sequential dependencies in time-series measurements. Conversely, the U-Net architecture shows potential for analyzing fluoroscopic images to identify abnormal joint mechanics, which could be adapted to classify HV-related structural changes [47]. The combination of LSTM for temporal prediction and U-Net for spatial feature extraction may facilitate proactive management of compensatory biomechanical patterns in HV patients, potentially enabling personalized rehabilitation planning. Similarly, postoperative rehabilitation following HV correction should incorporate systematic assessment and retraining of normal gait patterns to facilitate the unlearning of these established compensatory mechanisms.

There are several potential limitations to this study. First, although the subjects were all patients with bilateral HV, these populations were predominantly female, only a few male subjects were included in this study, which may limit the generalization of the conclusions. Despite inter-subject ratio differences, the higher prevalence [1] of HV in women (1:9) played a considerable role and therefore accounted for the different male/female ratios in this study. Additionally, a geriatric population could be added to the study as another age group likely to have problems with dynamic instability during gait. Second, the study did not consider the effect of symptom duration on compensatory mechanisms in patients with HV. Third, there is a known skin motion artifact related to motion capture technology, and the dual fluoroscopic imaging system is the most accurate noninvasive analysis system in sports medicine [41]; however, the system is more suitable for clinical applications due to its convenience and real-time nature based on a dual-headed infrared stereoscopic camera [14]. Fourth, only the ankle and adjacent knee joint kinematics were measured, not the electromyography activity of the associated muscles and articular kinetics. Fifth, unlike the ground gait, this study was unable to truly simulate the joint motion patterns of everyday gait even when walking at an adaptive speed in a running platform. Sixth, only walking was implemented at an adaptive speed as a daily activity for both groups, which may mask some differences that can only be observed at standardized speeds, and this did not involve more challenging maneuvers and movement patterns. Last, the lack of a force plate or using finite element modelling in the gait cycle to calculate and simulate the internal stresses of the joints associated with HV cannot reveal the propagation of deformation at the joint level. Combined with AI-based muscle activation analysis systems, this will enable the comprehensive assessment of motor compensation patterns and muscle synergies across diverse movement tasks in future clinical applications. These systems will facilitate the rapid, quantitative evaluation of injury risk factors, thereby providing robust empirical evidence to support evidence-based clinical decision-making frameworks.

## 5. Conclusions

In conclusion, this investigation provides the first comprehensive three-dimensional analysis of altered ankle and knee joint kinematics in patients with HV, establishing a biomechanical linkage between forefoot deformity and proximal joint compensations. It was found that HV pathology extends beyond localized foot mechanics, affecting the entire lower extremity kinetic chain and potentially increasing the risk of knee pathology, including medial compartment osteoarthritis and ligamentous injury. Therefore, patients with HV require a comprehensive lower extremity kinetic chain assessment rather than focusing solely on forefoot mechanics. The biomechanical framework established in this study provides a foundation for future investigations into the relationships between forefoot pathology and lower extremity function.

## Figures and Tables

**Table 1:** Description of demographic characteristics of the HV group and control group ^a^.

	HV Group	Control Group	*p* Value
N	23	23	
Age (Years)	36.9 ± 9.2	34.7 ± 5.8	0.783
Sex (n, Male/Female)	3/20	3/20	0.968
Height (cm)	161.9 ± 4.6	160.6 ± 5.0	0.344
Weight (kg)	58.0 ± 7.1	55.4 ± 6.4	0.212
Body mass index (kg/m^2^)	22.2 ± 2.8	21.5 ± 2.8	0.378

^a^ Values are expressed as mean ± SD.

**Table 2:** Differences in two-joint triple 6-DOF kinematics at initial contact ^a^.

	Variable	HV Group	Control Group	*p* Value
	N	23	23	
Ankle	Var-Val (+), deg	−0.54 ± 3.97	−3.40 ± 4.83	0.003 *
	Int-Ext (+), deg	−0.14 ± 3.60	−1.90 ± 4.30	0.035 *
	Ext-Fle (+), deg	−3.50 ± 4.94	−7.90 ± 4.44	<0.001 *
	Pos-Ant (+), mm	−0.06 (0.64) ^§^	0.11 (0.61) ^§^	0.078
	Med-Lat (+), mm	0.36 (0.78) ^§^	0.57 (0.62) ^§^	0.106
Knee	Var-Val (+), deg	−0.87 ± 3.20	0.53 ± 2.70	0.023 *
	Int-Ext (+), deg	1.96 ± 3.16	2.78 ± 5.42	0.39
	Ext-Fle (+), deg	9.59 ± 8.02	8.18 ± 6.27	0.35
	Pos-Ant (+), mm	0.69 ± 0.81	1.53 ± 0.52	<0.001 *
	Med-Lat (+), mm	0.26 ± 0.43	0.00 ± 0.70	0.036 *

^a^ Values are expressed as the mean ± SD, except for special descriptions. Var-Val, varus–valgus; Int-Ext, internal–external rotation; Ext-Flex, extension–flexion; Pos-Ant, posterior and anterior; Med-Lat, medial–lateral. ^§^ Values (failed normality tests) are shown as a median (interquartile range), with corresponding *p* values obtained by the Wilcoxon signed-rank test. * Statistically significant difference (*p* < 0.05), paired *t* test, two-tailed.

**Table 3:** Differences in two-joint triple six-degree-of-freedom kinematics at peak flexion of the stance phase.

	Variable	HV Group	Control Group	*p* Value
	N	23	23	
Ankle	Var-Val (+), deg	−4.01 ± 4.57	−4.20 ± 5.00	0.853
	Int-Ext (+), deg	−2.36 (6.52) ^§^	−5.05 (7.82) ^§^	0.043 ^§^
	Ext-Fle (+), deg	5.90 (12.18) ^§^	2.29 (7.12) ^§^	0.006 ^§^
	Pos-Ant (+), mm	−0.57 (0.61) ^§^	−0.41 (0.65) ^§^	0.128
	Med-Lat (+), mm	0.80 (0.53) ^§^	0.85 (0.72) ^§^	0.827
Knee	Var-Val (+), deg	−4.89 ± 5.60	−3.18 ± 4.60	0.112
	Int-Ext (+), deg	−1.22 (6.04) ^§^	1.37 (7.92) ^§^	0.009 ^§^
	Ext-Fle (+), deg	30.56 ± 11.98	29.6 ± 9.39	0.669
	Pos-Ant (+), mm	0.04 (0.83) ^§^	−0.05 (0.56) ^§^	0.809
	Med-Lat (+), mm	0.41 (0.70) ^§^	0.25 (0.80) ^§^	0.182

Var-Val, varus–valgus; Int-Ext, internal–external rotation; Ext-Flex, extension–flexion; Pos-Ant, posterior and anterior; Med-Lat, medial–lateral. ^§^ Values (failed normality tests) are shown as a median (interquartile range), with corresponding *p* values obtained by the Wilcoxon signed-rank test.

## Data Availability

The datasets generated and/or analyzed during the current study are not publicly available because the data are confidential patient data, but they are available from the corresponding author upon reasonable request.
